# Cultural Adaptation and Preliminary Assessment of the Italian Version of the FICA© Spiritual History Tool in the context of End-Of-Life Cancer Patients

**DOI:** 10.1007/s10943-026-02614-5

**Published:** 2026-03-08

**Authors:** Andrea Bovero, Arianna Scomazzon, Irene Di Girolamo, Chiara Lamannis, Christina Puchalski, Mario Cagna, Francesca Cotardo

**Affiliations:** 1 Clinical Psychology Unit, A.O.U. Città Della Salute e Della Scienza di Torino, Turin, Piedmont Italy; 2 Department of Clinical and Biological Sciences, University of Turin, Orbassano, Piedmont Italy; 3 Department of Medicine and Health Sciences, George Washington University School of Medicine, Washington, DC USA; 4 Asl4, Azienda Sociosanitaria Ligure N.4, Sistema Sanitario Regione Liguria, Chiavari, Liguria Italy

**Keywords:** Spiritual care, FICA© Spiritual History Tool, Italian adaptation, End-of-life cancer patients

## Abstract

Spirituality is widely recognized to play an important role in the experience of terminally ill cancer patients, and several studies have shown that spiritual dimensions in their care can significantly enhance their quality of life and emotional well-being. The FICA© Spiritual History Tool, developed by Dr. Christina Puchalski, provides clinicians with a framework for addressing patients’ spiritual histories. The aim of this study was to translate, culturally adapt, and apply the FICA© Spiritual History Tool in a sample of Italian end-of-life cancer patients and to identify the spirituality-related themes that emerge. The cultural adaptation process included forward–backward translations of the FICA© Spiritual History Tool, followed by its application through in-person conversations. Of the 95 eligible participants, 80 terminally ill cancer patients (84%) were included. A thematic analysis of the responses was conducted. Findings indicated that spirituality can provide a source of support for many Italian patients coping with terminal cancer. Spirituality was most frequently reflected with the Catholic faith. Some participants reported not belonging to any specific religious community; instead, they primarily relied on the support received from family and friends. The Italian version of the FICA© Spiritual History Tool is clear, understandable and focused on exploring spirituality and its relevance in end-of-life conversations. Healthcare providers (HCPs) should be informed about and encouraged to use this tool to gather essential information about their patients’ spiritual history and perspectives, supporting personalized care beyond their physical illness.

**Supplementary Information:**

The online version contains supplementary material available at 10.1007/s10943-026-02614-5.

## Introduction

### Spirituality in Health and Palliative Care

Although spirituality and religion are often considered synonymous, these concepts are non-interchangeable. According to Koenig et al. (2001), religion can be defined as “*an organized system of beliefs, practices, rituals and symbols designed to facilitate closeness to the sacred and the transcendent (God, Higher Power or Truth)*” based on traditions, rules, and cultural contexts (Damiano et al., 2016; Delgado-Guay, 2014; Demir, 2019; H. Koenig et al., 2001; H. G. Koenig, 2012; Panzini et al., 2017; Paul Victor & Treschuk, 2020; Reid, 2012).

Spirituality, instead, extends beyond institutional or denominational boundaries. Many individuals who identify as spiritual do not adhere to a particular religion, but cultivate a personal search for meaning, connection, and transcendence (Bovero et al., 2016; Panzini et al., 2017).

Spirituality is intrinsically linked to health and well-being, as the World Health Organization (WHO) has identified spirituality as the fourth dimension of health (Chen et al., 2018; Sepúlveda et al., 2002). In line with the WHO internationally accepted definition from the WHO (2023) and the European Association for Palliative Care (EAPC) (Best et al., 2020), spirituality can be described as *“the dynamic and intrinsic aspect of humanity through which persons seek ultimate meaning, purpose, and transcendence, and experience relationship to self, family, others, community, society, nature, and the significant or sacred.”* (NCP, 2018). This definition emphasizes spirituality as a universal human dimension expressed through beliefs, values, traditions, and practices that shape meaning and connection throughout life, illness, and at the end of life. Accordingly, spirituality has long been recognized as a key component of care for terminally ill cancer patients. Exploring spiritual needs at the end of life allows healthcare providers (HCPs) to gain deeper insight into patients’ values and priorities, enhance the therapeutic relationship, foster trust, and contribute to greater satisfaction with the care (Best et al., 2023a, 2023b; Steinhauser et al., 2017).

The 2014 WHO Resolution WHA67.19 (WHO, 2014) reaffirmed the spiritual dimension as an integral component of comprehensive palliative care and explicitly stated that addressing patients’ spiritual needs represents both an ethical obligation of HCPs and an ethical responsibility of the health system. More recently, the WHO defined palliative care as “*an approach that improves the quality of life of patients and their families facing the problems associated with life-threatening illness, through the prevention and relief of suffering by means of early identification and impeccable assessment and treatment of pain and other problems, physical, psychosocial and spiritual*” (Steinhauser et al., 2017; WHO, 2023). Over the past two decades, numerous studies have documented the association between spirituality, quality of life (QoL), and emotional well-being among terminally ill cancer patients (Hayden et al., 2022). In a comprehensive systematic review including 1,548 participants with terminal illness, Chen et al. (2018) found that spiritual care interventions, particularly those using a narrative approach, had a promising effect on QoL and spiritual well-being. Improvements were most evident in emotional and social domains. Furthermore, spiritual care interventions have been shown to alleviate existential suffering, promote adaptive coping, and help patients maintain a sense of meaning and dignity until the end of life (Bovero et al., 2016, 2019, 2023; Breitbart, 2002; Hayden et al., 2022; Sabet et al., 2023). By contrast, neglecting the spiritual dimension has been associated with lower QoL and with greater use of aggressive and expensive treatments (Gijsberts et al., 2019). Despite the widely acknowledged importance of the spiritual dimension of palliative care, spiritual well-being is frequently not adequately addressed by HCPs when caring for patients at the end of life (Matos et al., 2024). This gap may occur for several reasons such as lack of training in managing spiritual needs and limited time spent collecting relevant information (Best et al., 2023a, 2023b; Bovero et al., 2024; Sleeth et al., 2025). To address these barriers, several tools have been developed to help clinicians—including medical physicians and allied health practitioners (e.g. physical therapists, occupational therapists, speech language pathologists, spiritual care generalists, spiritual care specialists, and clinical social workers)—to engage in conversations about spirituality (Carey & Mathisen, 2018; Gijsberts et al., 2019; Saguil et al., 2012).

## *The *FICA©* Spiritual History Tool*

The FICA© Spiritual History Tool was specifically designed to guide clinicians in exploring patients’ spiritual histories. Developed by Dr. Christina Puchalski (Borneman et al., 2010; Puchalski, 2021), it is now maintained by the George Washington Institute for Spirituality and Health (GWISH). It provides clinicians with a framework for discussing spirituality issues with their patients. FICA© is an acronym for the four domains it touches on, namely **F**aith, Spiritual or beliefs, and what gives people meaning and purpose in their lives; the **I**mportance of spirituality for the individual and whether it impacts healthcare decision-making or how people care for themselves; the spiritual **C**ommunity which might be faith-based or other communities that matter most to patients, and interventions to **A**ddress spirituality needs, for example, referral to healthcare chaplains or supporting meditation or other practices that patients find helpful (Borneman et al., 2010).

The FICA© Spiritual History Tool has been widely used across diverse clinical settings, including serious illness and palliative care, and has demonstrated utility in improving communication about meaning, values, and spiritual distress among patients with advanced disease (Balboni et al., 2022; Borneman et al., 2010; Puchalski et al., 2014). Indeed, the questions can be flexibly adapted to different cultural and clinical contexts, and the person they are addressed to allows HCPs to personalize their communication on spiritual beliefs and needs (Balboni, 2024; Balboni et al., 2022).

Furthermore, clinicians are expected to act as spiritual care generalists, collaborating closely with spiritual care specialists, such as chaplains, to address patients’ spiritual concerns in daily clinical practice (Carey et al., 2026; Puchalski et al., 2009, 2014; Szilagyi et al., 2025).

### Aim of the Study

Although palliative care approaches have begun to emphasize spirituality, in Italy attention to the spiritual dimension remains limited, and challenges in addressing patients’ psycho-existential needs through a holistic model of care that transcends the traditional biomedical paradigm.

To foster conversations that integrate spiritual care experience into clinical practice routine, the present study aimed to translate and culturally adapt and apply the FICA© Spiritual History Tool within the Italian end-of-life palliative care context. Furthermore, the study explored the spirituality-related themes that emerge among patients with terminal cancer.

## Materials and Methods

### Study Design and Participants

The sample was recruited from September 2022 to April 2023 at Città della Salute e della Scienza di Torino University Hospital and Vittorio Valletta Hospice, in Turin, Italy. The study was approved by the Ethics Committee for the Hospital and Hospice; protocol number 0034403, procedure number CS2/1178. The sample consisted of cancer patients at the end of life. The inclusion criteria were: an age of 18 years or older; a cancer diagnosis; able to provide informed consent; meeting the criteria for receiving palliative care (National Law on Palliative Care and Pain Treatment, Legislation no. 38/2010); being in the terminal phase of cancer with no feasible or appropriate curative treatments; an estimated life expectancy of less than 4 months; and a Karnofsky Performance Status (KPS) of 50 or lower (Schag et al., 1984). Exclusion criteria were having a diagnosis of a severe psychiatric condition and having difficulties that would prevent the provision of informed consent or protocol completion.

During the initial consultation, the psychologist introduced the study to the patient and obtained their informed consent. The psychologist assessed the patient’s psychological state and spiritual needs, providing empathetic support and carefully attending to their individual concerns. The Italian version of the FICA© History Tool was then administered, with the responses recorded and subsequently transcribed verbatim by one of the researchers involved in the study.

### Measurements

Following the standard procedure for translating a scale into a different language proposed by Wild et al. (2005; Koenig et al., 2021), the original version of the FICA© tool (Borneman et al., 2010) was translated into Italian through forward–backward translation. This involved preparation, forward translation of the tool from its original language, in this case English, into Italian by an expert translator, followed by back-translation of the Italian version back into English by a different translator blind to the original version. The two English versions were reviewed and compared by the researchers to identify any problems or improvements to make in the translated (Italian) version.

The Italian version of the four open questions from the FICA© Spiritual History Tool is provided in Appendix.

These questions allow clinicians to assess both spiritual distress and spiritual resources of strength and patients to share their beliefs and concerns about spirituality. They assessed the following four domains of spirituality: the patient’s faith, beliefs, and sense of meaning and purpose; the importance of spirituality for the individual’s life and its influence on their healthcare decision-making; the individual’s spiritual community; and ways to address the patient’s spirituality, such as interventions or appropriate referrals to spiritual care specialists (Puchalski, 2021; Puchalski & Romer, 2000).

### Statistical Analysis

Statistical analysis was performed using the Statistical Package for Social Sciences (SPSS®) software version 25.0 for Windows®. Descriptive statistics were used to analyse the sociodemographic characteristics of the sample.

Thematic analysis of the final Italian adaptation of the FICA© Spiritual History Tool transcripts was conducted by three independent experts (F.C., I.D.G., and C.L.) to identify the central themes and sub-themes expressed by each patient. In the first phase of the analysis, the three researchers identified and labelled keywords that were repeatedly present in patients’ responses. Later, they compared the labels identified and selected a unique code for each theme that had emerged. Finally, the relative and per cent frequencies of these themes and sub-themes were calculated.

## Results

### Sociodemographic and Clinical Characteristics of the Sample

A total of 95 patients were invited to take part in the study; ten patients chose not to participate for personal reasons, two felt unable to answer the questions due to worsening disease, and three declined after reading the interview as they were uninterested. The final sample of the study consisted of 80 end-of-life cancer patients, all of whom met the inclusion criteria. The patients were predominantly women (61.3%), with an average age of 64.5(± 13.9) years. Most of the patients who took part in the research had metastatic cancer (63.6%), and the most common diagnosis was gastrointestinal cancer (18.8%). Of the patients interviewed, 85% self-identified as belonging to the Catholic Faith. More than half of the patients interviewed (67.5%) reported not to practice their religion, and only 32.5% reported to actively attend places of worship on a regular basis (Table 1).

**Table 1:** Participant clinical and sociodemographic characteristics (*N* = 80)

Variables	Frequency	Percentage
Sex		
Female	49	61.3
Male	31	38.8
Site		
Hospital	63	78.8
Hospice	17	21.3
Education		
Primary school	19	23.8
Middle school	18	22.5
High school	39	48.8
Graduate	4	5
Marital status		
Married	40	50
Single	20	25
Widow(er)	11	13.8
Divorced	9	11.3
Employment status		
Retired	46	57.5
Employed	19	23.8
Unemployed	8	10
Self-employed	7	8.8
Cancer site		
Gastrointestinal	15	18.8
Respiratory	14	17.5
Genitourinary	14	17.5
Hepatic/pancreatic	10	12.5
Head/neck cancer	7	8.8
Other	20	24.9
Cancer stage		
Local	17	21.25
Loco-regional	11	13.75
Metastatic	52	65
Religion		
Catholic	68	85
Orthodox	3	3.8
Muslim	2	2.5
Jehovah’s witness	2	2.5
Atheist	5	6.3
Practicing their religion		
Yes	26	32.5
No	54	67.5
Disease diagnosis and prognosis awareness		
No diagnosis or prognosis	3	3.8
Diagnosis only (not aware of prognosis)	20	25
Diagnosis and prognosis overestimation	16	20
Prognosis but no diagnosis	1	1.3
Total awareness	40	50

	Mean	Standard Deviation
Age	64.5	13.9
KPS	42.4	8.5

The sociodemographic and clinical characteristics of the sample are presented in Table 1.

### Qualitative Analysis of Italian Adaptation of the FICA© Spiritual History Tool

Tables 2, 3, 4, 5 present a summary of the key themes identified through thematic analysis of patients’ responses to the Italian adaptation of the FICA© Spiritual History Tool, with representative patient quotations.

**Table 2:** Quantitative summary of key issues in response to the “F—Faith/Belief/Sense of Meaning” themes

“Faith/Belief/Meaning and purpose” themes	Frequency^a^ (Percentage)^b^
Connection with God and prayer	55 (66.8%)
Faith as existential support	27 (33.8%)
Faith as strength/hope/outlet to face illness/loss	24 (30.0%)
Connection with transcendental figures (angels, saints, the deceased)	15 (18.8%)
Change in approach to Faith and prayer since illness onset	11 (13.8%)
Uncertain beliefs about faith	9 (11.3%)
Critical beliefs about faith	7 (8.8%)
Connection with the Church as an institution	3 (3.8%)
Faith as ethical teaching	1 (1.3%)

Examples

FICA©19: Let’s say that, in general, the Faith I once had is no longer there, perhaps because everyday things have now taken over and have somewhat diverted my attention away from spirituality. I think I have made religion more of a lifestyle. An example is my desire to help others. I am inclined to help others, perhaps due to my profession; in any case, I am convinced that there are different ways to be a doctor and that an extra word to a patient can be appreciated. The same applies to how I raise my children

FICA©21: I don’t even know how to explain my spirituality. I feel that I am helped not only by medicine in facing the illness but also by God, or perhaps by a guardian angel–I’m not really sure, but I believe there is something above that gives me a lot of strength. When I find myself in a moment of difficulty, I say a prayer, and this helps me a lot. It allows me to momentarily forget the problem I am facing and helps me calm down

^a^Number of participants who endorsed each theme

^b^Proportion of participants within the total sample (*N* = 80)

**Table 3:** Quantitative summary of the responses to the “I—Importance and Influence” themes

“Importance and Influence” themes	Frequency^a^ (Percentage)^b^
Spirituality as a support to face pain/illness and the related decisions	47 (58.8%)
Spirituality has no influence on health/illness	38 (47.5%)
Spirituality as providing strength/positive attitudes towards life	36 (45.0%)
Spirituality as a source of trust/hope/relief/well-being	18 (22.5%)
Spirituality as an intimate connection	6 (7.5%)
Spirituality as a duty	4 (5.0%)
Spirituality as moral support	3 (3.8%)
Hyper-involvement in spirituality	2 (2.5%)

Examples

FICA©19: I have generally been somewhat influenced by my belief in something. This influence extends not only to my health but also to my work. For example, it has helped me choose not to stop at just measuring the patient's blood pressure, but to add a bit of humanity, to see the person rather than just the patient in the bed. For instance, during inter-divisional night shifts, when someone passed away, while waiting for the death certificate, I would always say a prayer, like the Eternal Rest Prayer. I believe that being raised in a religious way has ingrained these teachings in me, they are a part of who I am

FICA©21: I think it influences me, at least to some extent. Let’s say that I believe in God, even though I also recognize the importance of medicine in treating my illness, and I feel that it helps me to some extent. And when I say my prayers, I feel good, as if I were liberated

^a^Number of participants who endorsed each theme

^b^Proportion of participants within the total sample (*N* = 80)

**Table 4:** Quantitative summary of key issues in response to the “C—Community” themes

“Community” key themes	Frequency^a^ (Percentage)^b^
Significant connections with family/friends/others	77 (96.3%)
Do not belong to a spiritual community	59 (73.8%)
Spirituality experienced individually rather than in a community	24 (30.0%)
Attend place of worship	14 (17.5%)

Examples

FICA©22: When I received my diagnosis, I didn’t keep it hidden from anyone, except initially from my parents and my youngest child. I really felt the need to share it, perhaps to seek support from all these people

FICA©67: I experience religion as a lifeline and participating in Mass on Sundays makes me feel part of a group, feel more supported and uplifted. Then there is my family. First and foremost, my wife. She is my oxygen; I couldn’t live without her. Then there are my parents and siblings; I care for them deeply, and they are a great support for me

^a^Number of participants who endorsed each theme

^b^Proportion of participants within the total sample (*N* = 80)

**Table 5:** Quantitative summary of key issues in response to the “A—Address/Action in Care” themes

“Address/Action in Care” themes	Frequency^a^ (Percentage)^b^
Not necessary	53 (66.3%)
Spirituality issues should be addressed by the medical team	14 (17.5%)
Healthcare providers are not responsible for addressing spirituality issues and they should not get involved	13 (16.3%)
Appreciate the availability of spiritual figures	5 (6.3%)
Spirituality provides a strategy for diverting attention away from the illness	2 (2.5%)

Examples

FICA©08: In reality, I have no expectations of the healthcare staff in that regard; I believe this is something where everyone must find their own inner balance. I expect the healthcare providers to take care of me, to look after my physical well-being. I do not have any expectations about the care of my spirit

FICA©79: In general, I would like healthcare providers to care for me on both a medical and human level, above all on the human level. I need someone who makes me feel heard, especially in those moments when I feel alone. ^a^Number of participants who endorsed each theme

^b^Proportion of participants within the total sample (*N* = 80)

“F–Faith, Beliefs and Meaning and purpose” Themes (Table 2). The most common responses referred to a sense of connection with God and prayer, reported by 55 patients (68.8%). This was followed by the view that faith represented an essential source of emotional and spiritual support in 27 patients (33.8%). Twenty-four patients (30.0%) described their faith as providing strength, hope, or helping them to cope with their cancer. In 19 participants (23.9%), faith was also viewed as providing ethical guidance and connection with transcendental figures (e.g. angels, saints, or the deceased) or with the Church as an institution. Eleven patients (13.8%) reported a change in their approach to faith and prayer since the onset of their illness. Finally, nine patients (11.3%) expressed uncertainty, and seven (8.8%) expressed critical beliefs regarding faith.

“I- Importance and Influence” Themes (Table 3). A total of 47 participants (58.8%) identified spirituality as a key source of support for dealing with pain, illness, and related decisions, making it the most salient theme of this subscale. Additionally, 36 (45.0%) viewed spirituality as offering strength or fostering positive attitudes towards life, while 18 (22.5%) described it as a source of trust, hope, relief, or well-being. Thirteen subjects exhibited an intense and pervasive involvement in spiritual beliefs or practices—for instance, daily rituals or prayers seen as moral duties or integral expressions of one’s identity—which occasionally extended beyond personal faith into relational or moral domains (e.g. praying for fellow patients who had passed away or feeling compelled to act according to religious teachings). This cluster included twelve patients that described spirituality as an intimate connection (7.5%), as a duty (5.0%), or as moral support (3.8%). In contrast, 38 participants (47.5%) reported that spirituality had no influence on their health or illness.

“C—Community” Themes (Table 4). This subscale explored whether patients identified spiritual communities, which might be faith-based or other supportive networks (e.g. family, secular communities). Although 96.3% reported feeling supported by family, friends, or other significant connections, 73.8% indicated not belonging to any formal spiritual community, experiencing their spirituality instead on an individual basis. Only 24 participants (30.0%) reported feeling part of a church’s community.

“A—Address/Action in Care” Themes (Table 5) explored how patients would like their spirituality to be integrated into their care. Fourteen subjects (17.5%) believed that spirituality-related issues should be considered by HCPs and expressed a desire for the clinical team to pay more attention to this aspect. Specifically, five patients (6.3%) mentioned that they would like to meet more frequently with a chaplain or a spiritual care provider, and two (2.5%) viewed spirituality as a welcome means of distraction from illness. Conversely, more than half of participants (*n* = 53; 66.3%) felt that spiritual care was not necessary for them, and 13 (16.3%) considered it outside the role of the medical team, as they viewed spirituality as something to be experienced independently of their medical care.

## Discussion

### Spirituality in the Italian Palliative Care context

Given the recognized role of spirituality as an essential aspect of whole-person health care in end of life (Bovero et al., 2016, 2019; Hayden et al., 2022; Sabet et al., 2023), the present study aimed to provide a practical tool for addressing spirituality by applying the Italian version of the FICA© Spiritual History Tool in the Italian palliative care context. The tool proved useful in helping patients to reflect on the meaning and purpose during their illness, discuss how spirituality influenced their self-care and health-related decisions, and identify sources of support, both spiritual and non-spiritual. It also gave patients the opportunity to share their spiritual resources, concerns, and preferences and to highlight any unmet needs or areas where further support might be desired, including from their HCPs. The flexibility of the tool makes the Italian adaptation of the FICA© a useful resource in palliative care, as it provides a patient-focused way for physicians or other HCPs to learn more about spiritual needs alongside patients’ personal history and standard clinical assessments.

### Comparison with Previous Literature and Cross-Cultural Perspectives

In line with the previous literature (Borneman et al., 2010) which emphasized the importance of spiritual care as an essential domain of quality patient care and endorsed the FICA© tool as a means of integrating spirituality into clinical assessment, our findings confirm that the Italian version is an acceptable tool for improving communication between clinicians and patients about spirituality, even in an end-of-life oncology setting.

In the American context of the FICA© (Borneman et al., 2010), the most frequent responses related to faith, beliefs, and meaning and purpose referred to an appreciation of family and life, followed by life activities, such as sense of purpose at work, friends, accomplishments, self-sufficiency, and productivity. By contrast, in our Italian sample, most patients identified themselves as religious and described experiencing spirituality primarily through a connection with God and prayer, confirming that strong link between spirituality and religion in the Italian context (Bovero et al., 2019). Furthermore, this pattern may also be partly influenced by the relatively advanced average age of our participants. Indeed, previous research suggests that non-religious or secular forms of spirituality are more common among the younger generations (Russo-Netzer, 2019).

### Clinical Implications

According to our results, end-of-life cancer patients considered spirituality and the acknowledgment of spiritual needs as a key source of support in dealing with pain, illness, and related decisions; dimensions that they felt should be addressed by the medical team. However, almost half of the sample perceived spirituality as having no influence on their health or illness or described it merely as a duty. Therefore, they did not express a need for spiritual assistance from their HCPs. The high proportion of patients belonging to traditional religiosity may have influenced the perception that details related to spiritual care during hospitalization should not be shared within healthcare teams. This finding highlights the importance for HCPs of considering aspects related to the patient’s unique preference for spiritual care, ensuring that they could feel supported for dealing with illness, in ways that are meaningful to them rather than relying only on assumptions based solely on religious background.

Although participants in our study expressed a strong sense of belonging to the Catholic faith, similar to the findings of the original American validation (Borneman et al., 2010), only a minority reported feeling actively involved in a spiritual community that provided support. Instead, most preferred a more individualized form of spirituality, drawing strength from their family, friends, and other significant relationships rather than from organized groups. This trend towards more individualized spirituality suggests that while religious affiliation remains significant, the sources of support patients rely on may be secular, emphasizing the importance of personal relationships over formal religious communities in providing not only emotional but also spiritual support.

Numerous studies have underscored the significant role of spirituality in enhancing patients’ QoL by reducing levels of depressive and anxiety symptoms and even influencing perceived physical well-being (Bovero et al., 2023; Ghiggia et al., 2021; Jim et al., 2015; Visser et al., 2010).

Future studies should adopt longitudinal designs to explore how integrated care, including sustained spiritual support such as chaplain involvement or targeted psychotherapeutic interventions such as Dignity Therapy (Chochinov et al., 2005) and Meaning-Centred Psychotherapy (Breitbart et al., 2010), may enhance cancer patients’ well-being at the end of life. Such approaches would allow examination of how spiritual needs and QoL evolve over time, providing insight into the role of continuous spiritual care. Furthermore, future research could carry out descriptive studies on larger populations and in different contexts, for example, by comparing the responses of outpatients and inpatients. Moreover, the original FICA© study combined qualitative and quantitative measures and concluded that FICA quantitative ratings were closely correlated with items from the QoL tools assessing aspects of spirituality (Borneman et al., 2010). The absence of quantitative data in our sample (e.g. mean importance scores or correlations with QoL and further psychological variables) limited our ability to assess the measurable impact of spirituality on patient outcomes, such as hopefulness, anxiety, or depression. It would also be valuable to investigate these relationships to clarify the reciprocal influence of spirituality, QoL, and overall well-being across different clinical populations.

In essence, this study suggested that the FICA© Spiritual History Tool might act as a bridge connecting patient spirituality and clinical care. It enables HCPs to explore patients’ spirituality, learning more about them as a person, beyond the standard biomedical care focused on treatments for terminal cancer. When spiritual distress is identified, HCPs should offer additional spiritual support by referring patients to a chaplain or trained spiritual care professional (Gilligan et al., 2017; Puchalski et al., 2014). This approach is consistent with the principles of lifestyle medicine perspective and holistic care, which encourages traditional approaches to patient care alongside deeper dimensions, such as spirituality (Faries et al., 2025). By integrating spiritual care as a core component into standard practice, the Italian FICA© Spiritual History Tool promotes patient-centred care and also strengthens collaboration between general-spiritual care providers, such as HCPs, and specialized spiritual care, provided by chaplains, ensuring continuity of support across the care pathway.

## Study Limitations

This study was conducted in a single end-of-life oncology cohort and excluded non-Italian-speaking patients as well as individuals who did not complete the FICA© Spiritual History. The thematic analysis aimed primarily to provide a descriptive and exploratory overview; therefore, the results are not statistically generalizable and may be influenced by small sample size and potential selection bias.

## Conclusion

Spiritual care is a key domain of high-quality palliative care, as recognized by international guidelines. The Italian version of the FICA© Spiritual History Tool represents a useful instrument for exploring patients’ spirituality and for gathering important clinical insights from end-of-life cancer patients in palliative care. It helps HCPs gain a deeper understanding of their patients, especially regarding aspects of their lives that extend beyond their physical symptoms and disease. The findings reinforce that addressing spiritual history in clinical settings is essential to enhancing dialogue between HCPs and their patients. A deeper understanding of the individual could foster the development of a stronger and more trusting patient–clinician relationship. Familiarity with the Italian adaptation of the FICA© and its use as a guide during consultations in end-of-life palliative care settings may also facilitate the integration of spirituality-related aspects into routine clinical conversation.

## Supplementary Information

Below is the link to the electronic supplementary material.

Supplementary file1 (DOCX 20 KB)

## References

[CR1] Balboni, T. A., VanderWeele, T. J., Doan-Soares, S. D., Long, K. N. G., Ferrell, B. R., Fitchett, G., Koenig, H. G., Bain, P. A., Puchalski, C., Steinhauser, K. E., Sulmasy, D. P., & Koh, H. K. (2022). “Spirituality in serious illness and health”: Correction. *JAMA: Journal of the American Medical Association,**328*(8), 780. 10.1001/jama.2022.11086 10.1001/jama.2022.11086 35819420

[CR2] Balboni, T. A. (2024). Spirituality in advanced cancer: Implications for care in oncologic emergencies. *Annals of Palliative Medicine,**13*(3), 568–574. 10.21037/apm-23-40 38834204 10.21037/apm-23-40

[CR3] Best, M. C., Jones, K., Merritt, F., Casey, M., Lynch, S., Eisman, J., Cohen, J., Mackie, D., Beilharz, K., & Kearney, M. (2023a). Australian Patient Preferences for the Introduction of Spirituality into their Healthcare Journey: A Mixed Methods Study. *Journal of Religion and Health,**62*(4), 2323–2340. 10.1007/s10943-022-01616-3 35918566 10.1007/s10943-022-01616-3 PMC9345780

[CR4] Best, M. C., Vivat, B., & Gijsberts, M.-J. (2023b). Spiritual Care in Palliative Care. *Religions*, *14*(3), 320. 10.3390/rel14030320

[CR5] Best, M., Leget, C., Goodhead, A., & Paal, P. (2020). An EAPC white paper on multi-disciplinary education for spiritual care in palliative care. *BMC Palliative Care,**19*(1), 9. 10.1186/s12904-019-0508-4 31941486 10.1186/s12904-019-0508-4 PMC6964109

[CR6] Borneman, T., Ferrell, B., & Puchalski, C. M. (2010). Evaluation of the FICA Tool for Spiritual Assessment. *Journal of Pain and Symptom Management,**40*(2), 163–173. 10.1016/j.jpainsymman.2009.12.019 20619602 10.1016/j.jpainsymman.2009.12.019

[CR7] Bovero, A., Gottardo, F., Tosi, C., Pidinchedda, A., Pesce, S., Botto, R., Caserta, M., Ostacoli, L., & Rossini, P. G. (2024). Spiritual issues, beliefs, needs, and resources in palliative healthcare providers: An Italian qualitative study. *Journal of Health Psychology*, 13591053241253046. 10.1177/13591053241253046 10.1177/13591053241253046 38738922

[CR8] Bovero, A., Leombruni, P., Miniotti, M., Rocca, G., & Torta, R. (2016). Spirituality, quality of life, psychological adjustment in terminal cancer patients in hospice. *European Journal of Cancer Care,**25*(6), 961–969. 10.1111/ecc.12360 26215314 10.1111/ecc.12360

[CR9] Bovero, A., Pesce, S., Botto, R., Tesio, V., & Ghiggia, A. (2023). Self-Transcendence: Association with Spirituality in an Italian Sample of Terminal Cancer Patients. *Behavioral Sciences,**13*(7), 559. 10.3390/bs13070559 37504006 10.3390/bs13070559 PMC10376349

[CR10] Bovero, A., Tosi, C., Botto, R., Opezzo, M., Giono-Calvetto, F., & Torta, R. (2019). The spirituality in end-of-life cancer patients, in relation to anxiety, depression, coping strategies and the daily spiritual experiences: A cross-sectional study. *Journal of Religion and Health,**58*(6), 2144–2160. 10.1007/s10943-019-00849-z 31165319 10.1007/s10943-019-00849-z

[CR11] Breitbart, W. (2002). Spirituality and meaning in supportive care: Spirituality- and meaning-centered group psychotherapy interventions in advanced cancer. *Supportive Care in Cancer,**10*(4), 272–280. 10.1007/s005200100289 12029426 10.1007/s005200100289

[CR12] Breitbart, W., Rosenfeld, B., Gibson, C., Pessin, H., Poppito, S., Nelson, C., Tomarken, A., Timm, A. K., Berg, A., Jacobson, C., Sorger, B., Abbey, J., & Olden, M. (2010). Meaning-centered group psychotherapy for patients with advanced cancer: A pilot randomized controlled trial. *Psycho-Oncology,**19*(1), 21–28. 10.1002/pon.1556 19274623 10.1002/pon.1556 PMC3648880

[CR13] Carey, L. B., Mathisen, B. A., Carey, C. L., Buckley, K. L., Malviya, S., & Drummond, D. (2026). Speech-language pathology, spirituality and palliative care. In L. Chahda, B. A. Mathisen, & L. B. Carey (Eds.), *Speech-Language Pathology and Palliative Care *(Chap. 10, pp. 195–232). Routledge. 10.4324/9781003241966-10

[CR14] Carey, L. B., & Mathisen, B. A. (Eds.). (2018). *Spiritual care for allied health practice: A person-centred approach*. Jessica Kingsley Publishers.

[CR15] Chen, J., Lin, Y., Yan, J., Wu, Y., & Hu, R. (2018). The effects of spiritual care on quality of life and spiritual well-being among patients with terminal illness: A systematic review. *Palliative Medicine,**32*(7), 1167–1179. 10.1177/0269216318772267 29708010 10.1177/0269216318772267

[CR16] Chochinov, H. M., Hack, T., Hassard, T., Kristjanson, L. J., McClement, S., & Harlos, M. (2005). Dignity therapy: A novel psychotherapeutic intervention for patients near the end of life. *Journal of Clinical Oncology,**23*(24), 5520–5525. 10.1200/JCO.2005.08.391 16110012 10.1200/JCO.2005.08.391

[CR17] Damiano, R. F., Costa, L. A., Viana, M. T. S. A., Moreira-Almeida, A., Lucchetti, A. L. G., & Lucchetti, G. (2016). Brazilian scientific articles on spirituality, religion and health. *Archives of Clinical Psychiatry (São Paulo),**43*(1), 11–16. 10.1590/0101-60830000000073

[CR18] Delgado-Guay, M. O. (2014). Spirituality and religiosity in supportive and palliative care. *Current Opinion in Supportive & Palliative Care,**8*(3), 308–313. 10.1097/SPC.0000000000000079 25029394 10.1097/SPC.0000000000000079

[CR19] Demir, E. (2019). The evolution of spirituality, religion and health publications: Yesterday, today and tomorrow. *Journal of Religion and Health,**58*(1), 1–13. 10.1007/s10943-018-00739-w 30523486 10.1007/s10943-018-00739-w

[CR20] Faries, M. D., Corrêa Fernandes, C., Phillips, E., West, T., & Stout, R. (2025). Religion and spirituality in lifestyle medicine. *American Journal of Lifestyle Medicine,**19*(2), 324–333. 10.1177/15598276241276770 39554923 10.1177/15598276241276770 PMC11562145

[CR21] Ghiggia, A., Pierotti, V., Tesio, V., & Bovero, A. (2021). Personality matters: Relationship between personality characteristics, spirituality, demoralization, and perceived quality of life in a sample of end-of-life cancer patients. *Supportive Care in Cancer,**29*(12), 7775–7783. 10.1007/s00520-021-06363-x 34169327 10.1007/s00520-021-06363-x PMC8550274

[CR22] Gilligan, T., Coyle, N., Frankel, R. M., Berry, D. L., Bohlke, K., Epstein, R. M., Finlay, E., Jackson, V. A., Lathan, C. S., Loprinzi, C. L., Nguyen, L. H., Seigel, C., & Baile, W. F. (2017). Patient–clinician communication: American Society of Clinical Oncology consensus guideline. *Journal of Clinical Oncology,**35*(31), 3618–3632. 10.1200/JCO.2017.75.2311 28892432 10.1200/JCO.2017.75.2311

[CR23] Gijsberts, M.H.E., Liefbroer, A. I., Otten, R., & Olsman, E. (2019). Spiritual care in palliative care: A systematic review of the recent European literature. *Medical Sciences,**7*(2), Article 25. 10.3390/medsci7020025 30736416 10.3390/medsci7020025 PMC6409788

[CR24] Hayden, L., Byrne, E., Deegan, A., Dunne, S., & Gallagher, P. (2022). A qualitative meta-synthesis examining spirituality as experienced by individuals living with terminal cancer. *Health Psychology Open,**9*(2), Article 205510292211215. 10.1177/20551029221121526 10.1177/20551029221121526 PMC9465615 36105766

[CR25] Jim, H. S. L., Pustejovsky, J. E., Park, C. L., Danhauer, S. C., Sherman, A. C., Fitchett, G., Merluzzi, T. V., Munoz, A. R., George, L., Snyder, M. A., & Salsman, J. M. (2015). Religion, spirituality, and physical health in cancer patients: A meta‐analysis. *Cancer,**121*(21), 3760–3768. 10.1002/cncr.29353 26258868 10.1002/cncr.29353 PMC4618080

[CR26] Koenig, H. G. (2012). Religion, spirituality, and health: The research and clinical implications. *ISRN Psychiatry,**2012*, 1–33. 10.5402/2012/278730 10.5402/2012/278730 PMC3671693 23762764

[CR27] Koenig, H.G., McCullough, M., & Larson, D. (2001). Handbook of religion and health. *Oxford University Press*. 10.1093/acprof:oso/9780195118667.001.0001

[CR28] Koenig, H. G., & Zaben, F. A. (2021). Psychometric validation and translation of religious and spiritual measures. *Journal of Religion and Health,**60*(5), 3467–3483. 10.1007/s10943-021-01373-9 34331172 10.1007/s10943-021-01373-9

[CR29] Matos, J., Querido, A., & Laranjeira, C. (2024). Spiritual care through the lens of Portuguese palliative care professionals: A qualitative thematic analysis. *Behavioral Sciences,**14*(2), Article 134. 10.3390/bs14020134 38392487 10.3390/bs14020134 PMC10886057

[CR30] NCP, National Consensus Project for Quality Palliative Care (2018). *Clinical Practice Guidelines for Quality Palliative Care* (4th edition). National Coalition for Hospice and Palliative Care. https://www.nationalcoalitionhpc.org/ncp-guidelines/10.1089/jpm.2018.0431 30179523

[CR31] Panzini, R. G., Mosqueiro, B. P., Zimpel, R. R., Bandeira, D. R., Rocha, N. S., & Fleck, M. P. (2017). Quality-of-life and spirituality. *International Review of Psychiatry,**29*(3), 263–282. 10.1080/09540261.2017.1285553 28587554 10.1080/09540261.2017.1285553

[CR32] Paul Victor, C. G., & Treschuk, J. V. (2020). Critical literature review on the definition clarity of the concept of faith, religion, and spirituality. *Journal of Holistic Nursing,**38*(1), 107–113. 10.1177/0898010119895368 31858879 10.1177/0898010119895368

[CR33] Puchalski, C. (2021). Spiritual Care in Health Care: Guideline, Models, Spiritual Assessment and the Use of the ©FICA Spiritual History Tool. In Spiritual needs in research and practice: The spiritual needs questionnaire as a global resource for health and social care. (Palgrave Macmillan, pp. 27–45). 10.1007/978-3-030-70139-0_3

[CR34] Puchalski, C. M., Vitillo, R., Hull, S. K., & Reller, N. (2014). Improving the spiritual dimension of whole person care: Reaching national and international consensus. *Journal of Palliative Medicine,**17*(6), 642–656. 10.1089/jpm.2014.9427 24842136 10.1089/jpm.2014.9427 PMC4038982

[CR35] Puchalski, C., Ferrell, B., Virani, R., Otis-Green, S., Baird, P., Bull, J., Chochinov, H., Handzo, G., Nelson-Becker, H., Prince-Paul, M., Pugliese, K., & Sulmasy, D. (2009). Improving the quality of spiritual care as a dimension of palliative care: The report of the consensus conference. *Journal of Palliative Medicine,**12*(10), 885–904. 10.1089/jpm.2009.0142 19807235 10.1089/jpm.2009.0142

[CR36] Puchalski, C., & Romer, A. L. (2000). Taking a spiritual history allows clinicians to understand patients more fully. *Journal of Palliative Medicine,**3*(1), 129–137. 10.1089/jpm.2000.3.129 15859737 10.1089/jpm.2000.3.129

[CR37] Reid, G. A. (2012). Spirituality and end of life issues: A review. *Journal of Religion, Spirituality & Aging,**24*(1–2), 120–130. 10.1080/15528030.2012.633054

[CR38] Russo-Netzer, P. (2019). “This is what real spirituality is all about”: A phenomenological exploration of the experience of spirituality outside institutional religion. *Psychology of Religion and Spirituality,**11*(4), 453–462. 10.1037/rel0000169

[CR39] Sabet, P., Karimi, S., Dehghan, A., & Bijani, M. (2023). Effect of spirituality-based palliative care on pain, nausea, vomiting, and the quality of life in women with colon cancer: A clinical trial in Southern Iran. *Journal of Religion and Health,**62*(3), 1985–1997. 10.1007/s10943-023-01742-6 36809520 10.1007/s10943-023-01742-6

[CR40] Saguil, A., Hospital, F. B. C., & Belvoir, F. (2012). The spiritual assessment. *American Family Physician,**86*(6), 546–550. 23062046

[CR41] Santos, C., & Michaels, J. L. (2022). What are the core features and dimensions of “spirituality”? Applying a partial prototype analysis to understand how laypeople mentally represent spirituality as a concept. *Psychology of Religion and Spirituality,**14*(1), 10–20. 10.1037/rel0000380

[CR42] Schag, C. C., Heinrich, R. L., & Ganz, P. A. (1984). Karnofsky performance status revisited: Reliability, validity, and guidelines. *Journal of Clinical Oncology,**2*(3), 187–193. 10.1200/JCO.1984.2.3.187 6699671 10.1200/JCO.1984.2.3.187

[CR43] Sepúlveda, C., Marlin, A., Yoshida, T., & Ullrich, A. (2002). Palliative care: The World Health Organization’s global perspective. *Journal of Pain and Symptom Management,**24*(2), 91–96. 10.1016/s0885-3924(02)00440-2 12231124 10.1016/s0885-3924(02)00440-2

[CR44] Sleeth, G., Gottlieb, P., Srinivasan, A., Thaddeus, U., Mennillo, M., & Anandarajah, G. (2025). Evaluation of the HOPE spiritual assessment model: A scoping review of international interest, applications and studies over 20+ years. *BMC Palliative Care,**24*(1), Article 191. 10.1186/s12904-025-01809-z 40624539 10.1186/s12904-025-01809-z PMC12236049

[CR45] Steinhauser, K. E., Fitchett, G., Handzo, G. F., Johnson, K. S., Koenig, H. G., Pargament, K. I., Puchalski, C. M., Sinclair, S., Taylor, E. J., & Balboni, T. A. (2017). State of the science of spirituality and palliative care research Part I: Definitions, measurement, and outcomes. *Journal of Pain and Symptom Management,**54*(3), 428–440. 10.1016/j.jpainsymman.2017.07.028 28733252 10.1016/j.jpainsymman.2017.07.028

[CR46] Szilagyi, C., Borchik, A. K., Twose, C., & Vandenhoeck, A. (2025). Chaplains’ collaboration and leadership in interprofessional healthcare: A scoping review Part 1: Multifaceted roles. *Journal of Health Care Chaplaincy,**26*, 1–31. 10.1080/08854726.2025.2563473 10.1080/08854726.2025.2563473 40999967

[CR47] Visser, A., Garssen, B., & Vingerhoets, A. (2010). Spirituality and well-being in cancer patients: A review. *Psycho-Oncology,**19*(6), 565–572. 10.1002/pon.1626 19916163 10.1002/pon.1626

[CR48] Wild, D., Grove, A., Martin, M., Eremenco, S., McElroy, S., Verjee-Lorenz, A., & Erikson, P. (2005). Principles of good practice for the translation and cultural adaptation process for patient-reported outcomes (PRO) measures: Report of the ISPOR Task Force for Translation and Cultural Adaptation. *Value in Health,**8*(2), 94–104. 10.1111/j.1524-4733.2005.04054.x 15804318 10.1111/j.1524-4733.2005.04054.x

[CR49] WHO. (2023). *Palliative care*. Geneva: World Heatlh Organization. https://www.who.int/europe/news-room/fact-sheets/item/palliative-care

