# Correction: Cultural Adaptation and Preliminary Assessment of the Italian Version of the FICA© Spiritual History Tool in the Context of End-of-Life Cancer Patients

**DOI:** 10.1007/s10943-026-02702-6

**Published:** 2026-06-20

**Authors:** Andrea Bovero, Arianna Scomazzon, Irene Di Girolamo, Chiara Lamannis, Christina Puchalski, Mario Cagna, Francesca Cotardo

**Affiliations:** 1 Clinical Psychology Unit, A.O.U. Città Della Salute e Della Scienza, Turin, Italy; 2 Department of Clinical and Biological Sciences, University of Turin, Regione Gonzole 10, 10043 Orbassano, TO Italy; 3 Department of Medicine and Health Sciences, George Washington University School of Medicine, Washington, CO USA; 4 Asl4, Azienda Sociosanitaria Ligure N.4, Sistema Sanitario Regione Liguria, Turin, Italy

**Correction to: Journal of Religion and Health** 10.1007/s10943-026-02614-5

In this article, a reference that had been masked during anonymized review was inadvertently not unmasked during the production process. The corrected text is as follows:

- Under the section Materials and Methods, subsection Study Design and Participants, the sentence: “The sample was recruited from September 2022 to April 2023 at [blinded as required by the journal] Hospital and the [blinded as required by the journal] Hospice” should read: “The sample was recruited from September 2022 to April 2023 at Città della Salute e della Scienza di Torino University Hospital and Vittorio Valletta Hospice, in Turin, Italy.”

In addition, the following corrections apply:

1. The residual table-placement instruction beneath Table 1—*“[before ‘Qualitative Analysis of Italian Adaptation of the FICA© Spiritual History Tool]”*—should be removed.
2. In the *Ethics Approval* section, the sentence: *“The present study was approved by the [blinded as required by the journal]”* should read: *“The present study was approved by the Ethics Committee for the Hospital and Hospice.”*

