# Supplementary material for: Cultural Adaptation and Preliminary Assessment of the Italian Version of the FICA© Spiritual History Tool in the context of End-Of-Life Cancer Patients
Source: J Relig Health. 2026 Mar 8;65(4):3681–96. doi: 10.1007/s10943-026-02614-5 (PMC13481408; doi:10.1007/s10943-026-02614-5)
Supplement: Supplementary file 1 [file 10943_2026_2614_MOESM1_ESM.docx]

**Appendix**. Italian version of the questions in the Italian adaptation of the FICA© Spiritual History Tool.

Italian adaptation of the FICA© Spiritual History Tool

**F – Fede, Credenze e Significato**

**F – Faith, Belief, Meaning**

- Si considera una persona spirituale o religiosa?
- Do you consider yourself to be spiritual? or Is spirituality something important to you?

- Ha convincimenti spirituali che La aiutano a gestire lo stress e i momenti difficili?
- Do you have spiritual beliefs, practices, or values that help you to cope with stress, difficult times, or what you are going through right now?

- Cosa dà senso/significato alla Sua vita?
- What gives your life meaning?

**I – Importanza e Influenza**

**I – Importance and Influence**

- Quale importanza ha la spiritualità nella Sua vita?
- What importance does spirituality have in your life?

- La Sua spiritualità ha influenzato il modo in cui si prende cura di se stesso, della Sua salute?
- Has your spirituality (or sources of meaning) influenced how you take care of yourself, particularly regarding your health?

- La Sua spiritualità La influenza nel prendere decisioni relative alla salute?
- Does your spirituality affect your healthcare decision making?

**C – Comunità**

**C – Community**

- Fa parte di una comunità di natura spirituale?
- Are you part of a spiritual community?

- Questo Le è di supporto/sostegno e come?
- As your community of support to you and how?

- C’è un gruppo di persone che Lei realmente ami o che siano importanti per Lei?
- Is there a group of people you really love or who are important to you?

**I – Intervento nell’assistenza alla persona**

**A – Address/Action in Care**

- In quale modo gradirebbe che io, il Suo operatore sanitario, soddisfacessi tali questioni nella cura della Sua salute?
- How would you like me, as your healthcare provider, to address spiritual issues in your healthcare?

**Note:** To accompany the non-English version of the scale, the English version of each item is provided below each Italian sentence.
